# Network embedding framework for driver gene discovery by combining functional and structural information

**DOI:** 10.1186/s12864-023-09515-x

**Published:** 2023-07-29

**Authors:** Xin Chu, Boxin Guan, Lingyun Dai, Jin-xing Liu, Feng Li, Junliang Shang

**Affiliations:** School of Computer Science, Qufu Normal University, Rizhao, 27826 China

**Keywords:** Driver gene, Gene interaction network, Network embedding, Mutation data, Classification algorithm

## Abstract

Comprehensive analysis of multiple data sets can identify potential driver genes for various cancers. In recent years, driver gene discovery based on massive mutation data and gene interaction networks has attracted increasing attention, but there is still a need to explore combining functional and structural information of genes in protein interaction networks to identify driver genes. Therefore, we propose a network embedding framework combining functional and structural information to identify driver genes. Firstly, we combine the mutation data and gene interaction networks to construct mutation integration network using network propagation algorithm. Secondly, the struc2vec model is used for extracting gene features from the mutation integration network, which contains both gene's functional and structural information. Finally, machine learning algorithms are utilized to identify the driver genes. Compared with the previous four excellent methods, our method can find gene pairs that are distant from each other through structural similarities and has better performance in identifying driver genes for 12 cancers in the cancer genome atlas. At the same time, we also conduct a comparative analysis of three gene interaction networks, three gene standard sets, and five machine learning algorithms. Our framework provides a new perspective for feature selection to identify novel driver genes.

**Supplementary Information:**

The online version contains supplementary material available at 10.1186/s12864-023-09515-x.

## Introduction

Cancer is one of the main causes of morbidity and mortality of human beings and seriously endangers human healthy [1]. It is caused by some somatic mutations, which destroy the normal growth of cells, leading to abnormal proliferation and tumor development [2]. The Cancer Genome Atlas (TCGA) [3] and the International Cancer Genome Consortium (ICGC) [4] have generated and evaluated cancer genetic data [5]. The key challenge in cancer genomics is to analyze, utilize and integrate this information in the most effective and meaningful way, which can contribute to the development of cancer biology directions and then translate this knowledge into clinical practice that can help a larger number of people [6, 7]. It plays a causal role in the occurrence or development of cancer, which is called "driver" mutation [8]. One of the main goals of cancer research is to identify all genes carrying mutations, which can drive the carcinogenesis in different tumor types [9]. However, the analysis of individual omics data is limited to exploring the underlying surface biological mechanisms and can only explain their molecular domains in isolation [10]. As a result, combining gene functional and structural information at different levels can help researchers better comprehend overall disease changes, with significant implications for cancer analysis, diagnosis, and treatment [11].

In the last decade, researchers have proposed several methods to identify potential cancer driver genes based on some typically commonly used public data. Among them, somatic mutations are very effective and are almost the basic type of data for prioritizing driver genes. In general, the easiest way to identify driver genes is to classify mutated genes according to the recurrence of cancer. In other words, the most frequent mutations are more likely to be the driver [12] and use the background mutation rate to identify the genes with significant mutations. Many calculation methods based on mutation frequency identification have been widely used in driver mutations and driving genes, such as MutSigCV [13]. Based on recurrence information, MutSigCV considers heterogeneity from three aspects of sample, gene, and mutation type, and assumes that background mutation rates are inconsistent for each cancer type. OncodriveFML detects both coding and non-coding cancer drivers by analyzing the functional impact of gene alterations [14]. Two-stage-vote based on mutation information, gene networks, and voting methods, the ensemble model is developed to identify driver genes of 33 kinds of cancer [15]. DriverML [16] uses supervised machine learning to analyze the functional effects of mutations to identify cancer drivers. MoProEmbeddings developed an innovative node embedding program to achieve the supervised classification of cancer driver genes through an unsupervised process [17]. deepDriver is proposed by performing convolution on mutation-based features of genes and their neighbors in the similarity networks. The method allows the convolutional neural network to learn information within mutation data and similarity networks simultaneously, which enhances the prediction of driver genes [18]. But cancer drivers in many methods will not be found because they are highly heterogeneous in the population [19]. The above method only takes into account the adjacent genes and does not take into account the information between genes that are far away. Most approaches identify driver genes based on the characteristics of genes surrounding or closer to the node [17].

The main objective of this study is to propose the incorporation of topological information into gene interaction networks that are more likely to contribute to the identification of cancer driver genes. Topological information is structural information, and if two nodes are in the same order, they are more similar in structure. In this work, we propose a network embedding framework that combines functional and structural information to discover driver genes. Firstly, the mutated genes are combined with the protein interaction network to construct the mutation integration network using the network propagation algorithm. Secondly, the struc2vec model is used for extracting gene features from the mutation integration network, which can find gene pairs with long-distance but similar structures. Therefore, the gene features have more comprehensive information, which contains both gene's functional and structural information and is more conducive to identifying potential cancer driver genes. Finally, machine learning algorithms are utilized to predict genes, and the top-ranked mutated genes are considered as the driver genes. In this paper, useful functional and structural information is extracted from mutation data and gene interaction networks. We performed a comprehensive evaluation of the framework based on TCGA data on somatic mutations in 12 cancers, using three well-known cancer gold standards sets for comparison, such as Cancer Gene Census(CGC) [20], Network of Cancer Genes (NCG) [21] and Integrative Onco Genomics (IntOGen) [22]. We also compare the framework with other methods and analyze the cancer driver genes identified by the framework.

## Material and methods

### Mutation data representation

The TCGA data included 12 cancer types with 11,565 genes from 3,110 samples, and Table 1 details the number of samples contained in each cancer type. This data is from the TCGA website (https://portal.gdc.cancer.gov/) and the Catalogue of Somatic Mutations in Cancer (COSMIC) [23].

**Table 1:** 12 cancer types and corresponding sample numbers

Cancer types		Patient number
BLCA	Bladder urothelial carcinoma	87
BRCA	Breast invasive carcinoma	763
COAD	Colon adenocarcinoma	89
GBM	Glioblastoma multiforme	290
HNSC	Head and neck squamous cell carcinoma	293
KIRC	Kidney renal clear cell carcinoma	417
LAML	Acute Myeloid Leukemia	195
LUAD	Lung adenocarcinoma	186
LUSC	Lung squamous cell carcinoma	160
READ	Rectum adenocarcinoma	104
OV	Ovarian serous cystadenocarcinoma	316
UCEC	Uterine corpus endometrial carcinoma	210

### Gene interaction network reconstruction

Three protein–protein interaction networks are used in this paper: HINT + HI2012 [24], iRefIndex [25] and InBio Map PPI network [26]. Hint + hi2012 combines the hint network and hi-2012, a group of protein–protein interactions, consisting of 40,783 interactions among 10,008 proteins. InBio Map PPI network has collected the data information of histone protein interaction, consisting of 612,997 interactions among 17,429 proteins. This large-scale data resource has been able to clarify the impact of multiple genes on disease development. IRefIndex is calculated by processing protein interaction records from databases such as BIND, BioGrid, and IntAct, among others, consisting of 91,872 interactions among 12,338 proteins.

### Cancer gene benchmarks

In the absence of a universally accepted gold standard set, it is difficult to determine which predicted genes performed well and which predictive tools performed adequately in previous studies [23, 27, 28]. To provide a comprehensive evaluation of our approach, several benchmark measurements were used to evaluate known driving datasets, Such as CGC, NCG, IntOGen. The CGC database manually compiled a list of 723 commonly used genes whose mutations have a causal link to cancer. It is generally accepted that a higher percentage of predictions in a CGC database indicates better performance. Apart from the CGC database, we also consider the NCG 7.0 database, which contains 2757 cancer genes from manually curated articles. Aside from these two datasets, the IntOGen database recently announced a fresh batch of 568 driver genes. A benchmark for cancer driver genes is overlap with the CGC, NCG, and IntOGen gene lists.

In this work, we use the mutation matrix ${\text{A}}$ to represent the mutation data of cancer types, which is a binary matrix with m samples as rows and n genes as columns, respectively. We use three reference gene interaction networks that all treat them in the same way. The mutation matrix and sequence are integrated with the protein network and put into our framework for operation.

## Methods

**Fig. 1:**
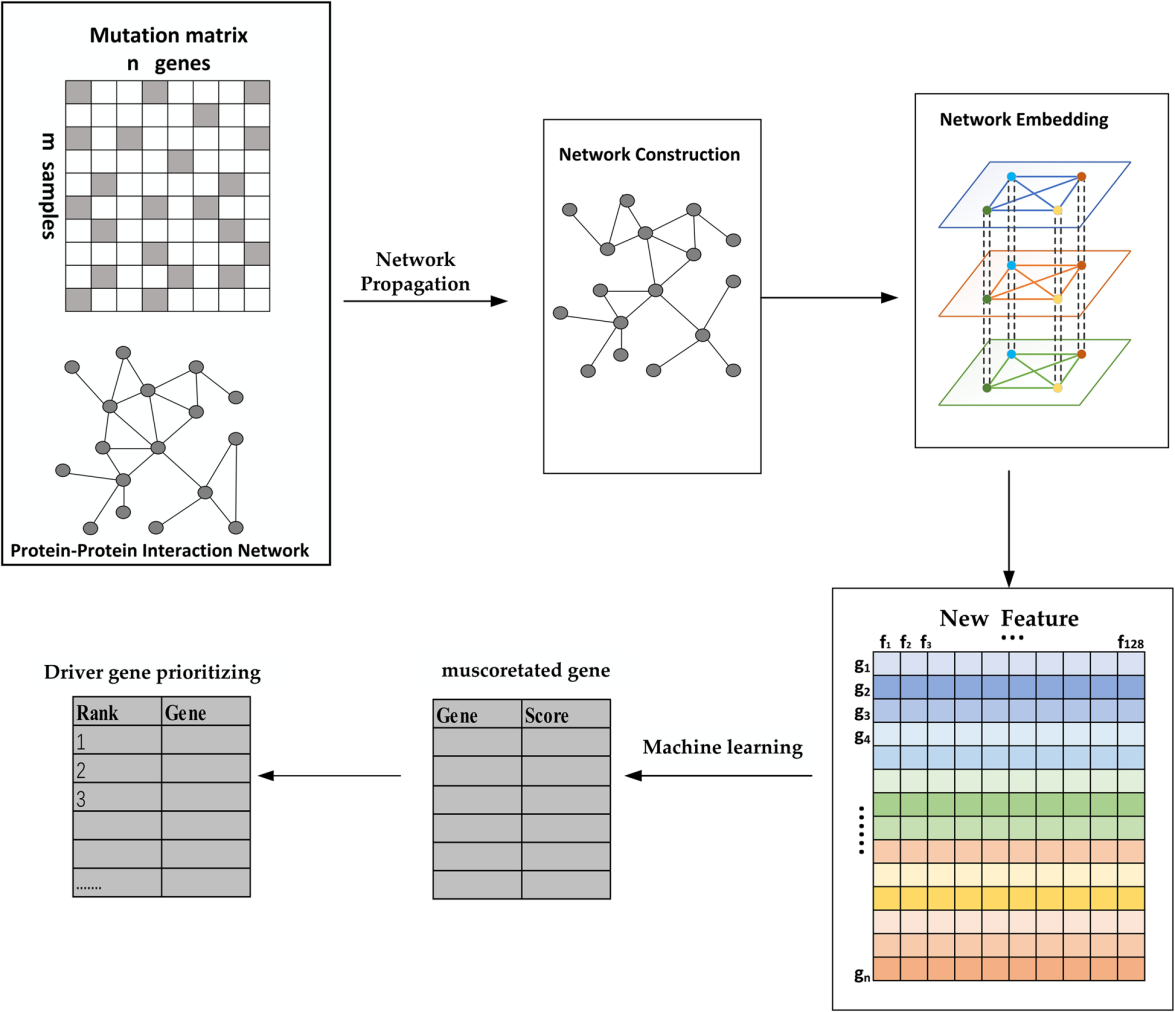
Overview of the network embedding framework by considering functional and structural information to identify driver genes

In this work, the network propagation approach [29] and the struc2vec [31] model are used as the basis. We propose a network embedding framework that combines functional and structural information to identify driver genes. We combine the mutated gene and protein–protein interaction network to construct a mutation integration network using a network propagation algorithm. Then, the struc2vec model is used to extract functional and structural information of genes from the mutation integration network. Finally, we learn the constructed features by machine learning method, and the top-ranked mutated genes are considered as the driver genes. To identify more cancer driver genes, an overview of our approach is shown in Fig. 1.

### Constructing mutation integration network

In this work, we integrate the mutant gene and protein interaction network and use network propagation embedding to overcome population-level heterogeneity. Because network propagation can enlarge the weak similarity between genes in protein networks of different patients [29]. This functional similarity can successfully obtain the functional link between the driver gene and any mutation, especially if the gene has a small number of mutations.

We use the network propagation algorithm to smooth the effect of mutation on the protein–protein interaction network of each sample [30]. For sample $s \in S$, the network propagation function is a random traversal based on the following function:

1 $${F}{\prime}=a{W}{\prime}{F}^{t-1}+\left(1-a\right)Y$$

where ${\text{F}}^{0} = {\text{Y}}$ is a row of the mutation matrix $A$ corresponding to $s$,${\text{t}}$ represents the time of update iteration, $Y$ represents a vector of gene expression for sample $s$.${\text{W}}{\prime}$ is the protein–protein network as a degree adjusted adjacency matrix. $\alpha$ is a parameter that regulates the similarity between networks. The network propagation process is carried out iteratively until $F^{{\text{t}}}$ converges, and the convergence condition is $||F^{t} - F^{t - 1} ||_{2} < 10^{ - 6}$. The resulting matrix $F^{{\text{t}}}$ is the propagated mutation profile for the sample $s$.

Our model runs with an unweighted network, which is obtained by cutting a threshold of similarity score. The threshold α for cutting the similarity score is discussed with the step size of 0.1. Then we consider the similarity score with the largest precision for detecting driver genes as the threshold. At last, 0.5 is selected as the threshold, which means the edges between each two gene with similarity score > 0.5 are reserved. And the detailed discussion is in the Supplementary 1.

### Network embedding

In this work, nodes in similar networks represent genes, and edges represent that the two genes have similar relationships. To better mine the characteristics of genes in the network, we use the network embedding method to learn a vector to represent the genes in the network. Node2vec model is a classical network embedding method, but it has a fatal disadvantage [31]. It is unable to effectively simulate long-distance nodes with structural similarities due to the restricted sampling length of walking. In order to overcome this shortcoming, we adopt the struc2vec model for the vectorization process of the newly constructed network nodes [32]. The Struct2vec model encodes structural similarity by constructing multilayer graphs to generate structural contexts for nodes. Compared with most algorithms, it can find gene pairs with long-distance but similar structures. Therefore, the struc2vec model is used for extracting gene features, the gene features have more comprehensive information, which contains both genes functional and structural information and is more conducive to identifying potential cancer driver genes. As a general rule, two nodes are more similar in structure if they have the same order. In other words, the structure of the two nodes should be more similar if all neighboring nodes of both nodes also have the same degree.

The structural similarity of node *x* and node $y$ is defined as follows:

2 $${f}_{k}\left(x,y\right)={f}_{k-1}\left(x,y\right)+g\left(s\left({R}_{k}\left(x\right)\right),s\left({R}_{k}\left(y\right)\right)\right)$$

where $R_{k} \left( x \right)$ is the set with a distance of $k$ from the node $x$, and $s\left( {R_{k} \left( x \right)} \right)$ represents the ordered sequence, $R_{k} \left( x \right)$ arranged according to the degree of nodes. $g\left( {s\left( {R_{k} \left( x \right)} \right),s\left( {R_{k} \left( y \right)} \right)} \right) > 0$ is the distance function that measures $s\left( {R_{k} \left( x \right)} \right)$ and $s\left( {R_{k} \left( y \right)} \right)$, and $f_{ - 1} = 0$.

Since the sizes of $s\left( {R_{k} \left( x \right)} \right)$ and $s\left( {R_{k} \left( y \right)} \right)$ are different, we use Dynamic Time Warping (DTW) [33] to measure the distance between the two sequences:

3 $$g\left( {s\left( {R_{k} \left( x \right)} \right),s\left( {R_{k} \left( y \right)} \right)} \right) = \frac{{\max \left( {s\left( {R_{k} \left( x \right)} \right),s\left( {R_{k} \left( y \right)} \right)} \right)}}{{\min \left( {s\left( {R_{k} \left( x \right)} \right),s\left( {R_{k} \left( y \right)} \right)} \right)}} - 1$$

The similarity of degree distributions among all node pairs in the network is calculated, and the similarity is used to generate a multilayer weighted graph. The edge weight between nodes $x$ and $y$ in the same layer is defined as:

4 $$\omega_{k} (x,y) = e^{{ - f_{k} (x,y)}} ,k = 0,1, \cdots ,k^{*}$$

where $k^{*}$ is the diameter of a similar network. The same nodes belonging to different levels are connected by directed edges. For each node $x$ in the $k$ layer, it is connected to $k - 1$ and $k + 1$ layers. The weight of the edge between different layers is defined as:

5 $$\omega \left( {x_{k} ,x_{k - 1} } \right) = \log (\Gamma_{k} (x) + e)$$

where $\Gamma_{k} (x)$ is the number of edges connected to $x$ in layer $k$ and the weight is greater than the average weight.

We use the biased random walk to carry out random walk in the weighted multi-layer graph to generate a node sequence. It is assumed that the walk takes place in the current layer with the probability of $q$ and jumps to other layers with the probability of $\left( {1 - q} \right)$. If it is determined to walk in the current layer, let it be in the layer $k$, then the probability from node $x$ to node $y$ is defined as:

6 $$p_{k} \left( {x,y} \right) = \frac{{e^{{ - f_{k} \left( {x,y} \right)}} }}{{Z_{k} \left( x \right)}}$$

where $z_{k} (x) = \sum\nolimits_{y \ne x} {e^{{ - f_{k} (x,y)}} }$ is the normalization factor of a node $x$ in the k-layer.

Through the random walk algorithm, each sampling gene is more inclined to select genes with highly similar gene structures to the current. If the jump is made, the probability of jumping $k + 1$ and $k - 1$ is as follows:

7 $$p_{k} (x_{k} ,x_{k + 1} ) = \frac{{\omega (x_{k} ,x_{k + 1} )}}{{\omega (x_{k} ,x_{k + 1} ) + \omega (x_{k} ,x_{k - 1} )}}$$

8 $$p_{k} (x_{k} ,x_{k - 1} ) = 1 - p_{k} (x_{k} ,x_{k + 1} )$$

In this study, we begin at the bottom layer and travel through the randomly chosen nodes. the length of each random walk sequence is set to 80, and each node generates 20 random walk sequences. we embedded each gene as a 128-dimensional vector. When generating the node sequence, the skip-gram model [34] is used to train the node sequence.

### Detection method

To better identify potential cancer driver genes, we integrate mutant gene vectors. For patients with different types of cancer, we predict genes based on structurally similar and functionally identical features. For each patient, we take the mutant gene vector to generate a new 128-dimensional feature. Then we use five machine learning algorithms including K-Nearest Neighbor (KNN) [35], Logistic Regression(LR) [36], XGBoost(XGBT) [37], Support Vector Machine(SVM) [38], and Random Forest(RF) [39] to predict cancer driver genes. We find that the XGBoost approach is the best.

XGBoost is a gradient boosting decision in which each time a tree is added, a new function is learned to fit the residuals of the previous prediction. After training is complete, each tree learns a correlation score based on the properties of the driving genes. Finally, the scores of each tree are simply summed to obtain the predicted value based on the target function gene. The objective function of XGBoost(XGBT) is as follows:

9 $${\text{L}}\left( \phi \right) = \sum\limits_{i} {l\left( {y_{i}{\prime} - y_{i} } \right)} + \sum\limits_{k} {\left( {\gamma T + \frac{1}{2}\lambda \sum\nolimits_{j = 1}^{T} {w_{j}^{2} } } \right)}$$

where $T$ is the number of leaves in the tree, ${\text{y}}$ is the label, $l$ is the module square of the score,$w$ of the leaf node in the tree.

### Fivefold cross-validation

Cross-validation is an evaluation method that aims to obtain a stable result. Therefore, we process datasets using fivefold cross-validation to create a stable and dependable supervised prediction model. In this work, to address the problem of unbalanced cancer gene datasets, we replaced oversampling, which tends to cause overfitting, with undersampling. Oversampling is not used because it is prone to over-fitting. Therefore, we repeatedly did undersampling 10 times to make effective use of the data. 80% of the genes are randomly selected as the training set and the remaining 20% as the test set. The average of the results of 100 runs is the final result. Through experiments, among 32 dimensions, 64 dimensions, and 128 dimensions, we find that the model learning 128 dimensions features has the best performance.

### Evaluation metrics

We use three gene standard sets to identify cancer driver genes. Evaluate the model's performance using five-folded cross-validation tests and a variety of commonly used metrics, including Receiver Operating Characteristic AUC (ROC-AUC), accuracy, precision, recall, specificity, and the F1 metric. The AUC value, namely the area under the receiver operating characteristic (ROC) curve, was selected as the evaluation index to judge the classification performance. We calculated the true positive rate (TPR) and the false positive rate (FPR) by changing the threshold to obtain the ROC curve. Several indicators are introduced below.

10 $$Accuracy = \frac{TP + TN}{{TP + FP + TN + FN}}$$

11 $$Precision = \frac{TP}{{TP + FP}}$$

12 $$Recall = \frac{TP}{{TP + FN}}$$

13 $$F1 - score{ = }2\frac{Precision \times Recall}{{Precision + Recall}}$$

ROC curve according to the following equation:

14 $$\left\{ {\begin{array}{*{20}c} {TPR = \frac{TP}{{TP + FN}}} \\ {FPR = \frac{FP}{{TN + FP}}} \\ \end{array} } \right\}$$

where True Negative (TN), True Positive (TP), False Negative (FN), and False Positive (FP), respectively, are in Eqs. 10– 14.

## Results

In this paper, we comprehensively evaluate mutation data from all 12 cancers from TCGA using multiple benchmark metrics, and we also perform individual analyses for each cancer. First, we compare the impact of data with and without mutation signatures on identifying driver genes. Then, we analyze the gold benchmark driver set and other algorithms from two different perspectives. We perform enrichment analysis on the detected genes to verify their biological functions. In addition, we summarize the new list of predictive driver genes and study several of them in depth.

### Comparison of algorithms in pan-cancer

We identify cancer driver genes from the whole pan-cancer through five models to discuss which model performed better. Discuss and analyze according to the data downloaded from TCGA. We used CGC as the gene standard set because the general CGC dataset is commonly used by everyone. The HINT + HI2012 network is integrated with mutation data and incorporated into our framework to predict potential cancer driver genes [23].

**Fig. 2:**
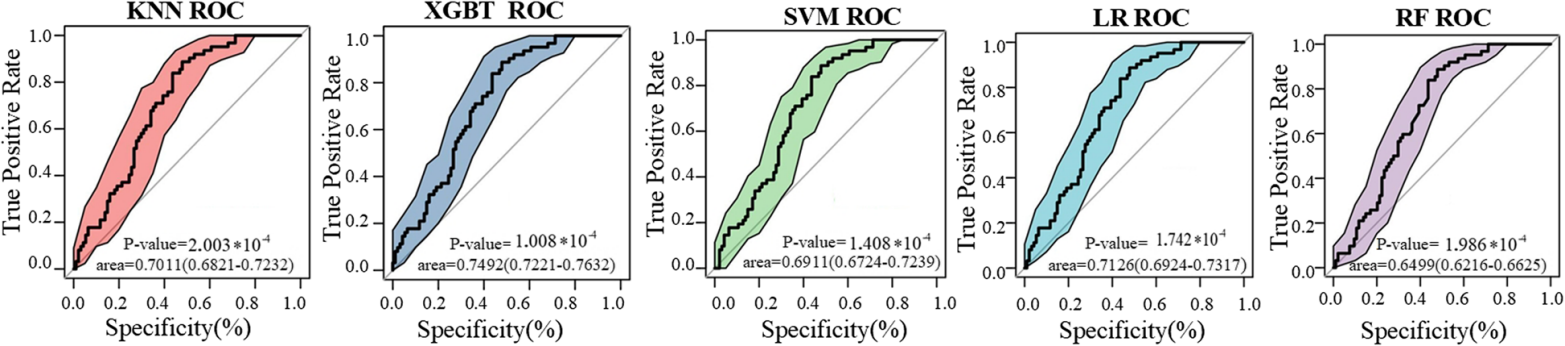
Comparison of ROC curves of five machine learning algorithms in the whole pan-cancer

As shown in Fig. 2, our framework analyzes the ROC curves of five models. ROC curve can easily find out the recognition ability of cancer-driver genes at any limited value. We run 100 fivefold cross-validations and averaged the results to get the final AUC value. In the same coordinate axis, by calculating the area under the ROC curve of each experiment, it is clear to see from Fig. 2 that the area under the ROC curve of XGBT is as large as 0.7492, which is better than the other four machine learning models. In other metrics, XGBT is higher than the other methods, except in the F1-score, which is slightly lower than the other methods (Table S1).

### Comparison of algorithms in each cancer

**Fig. 3:**
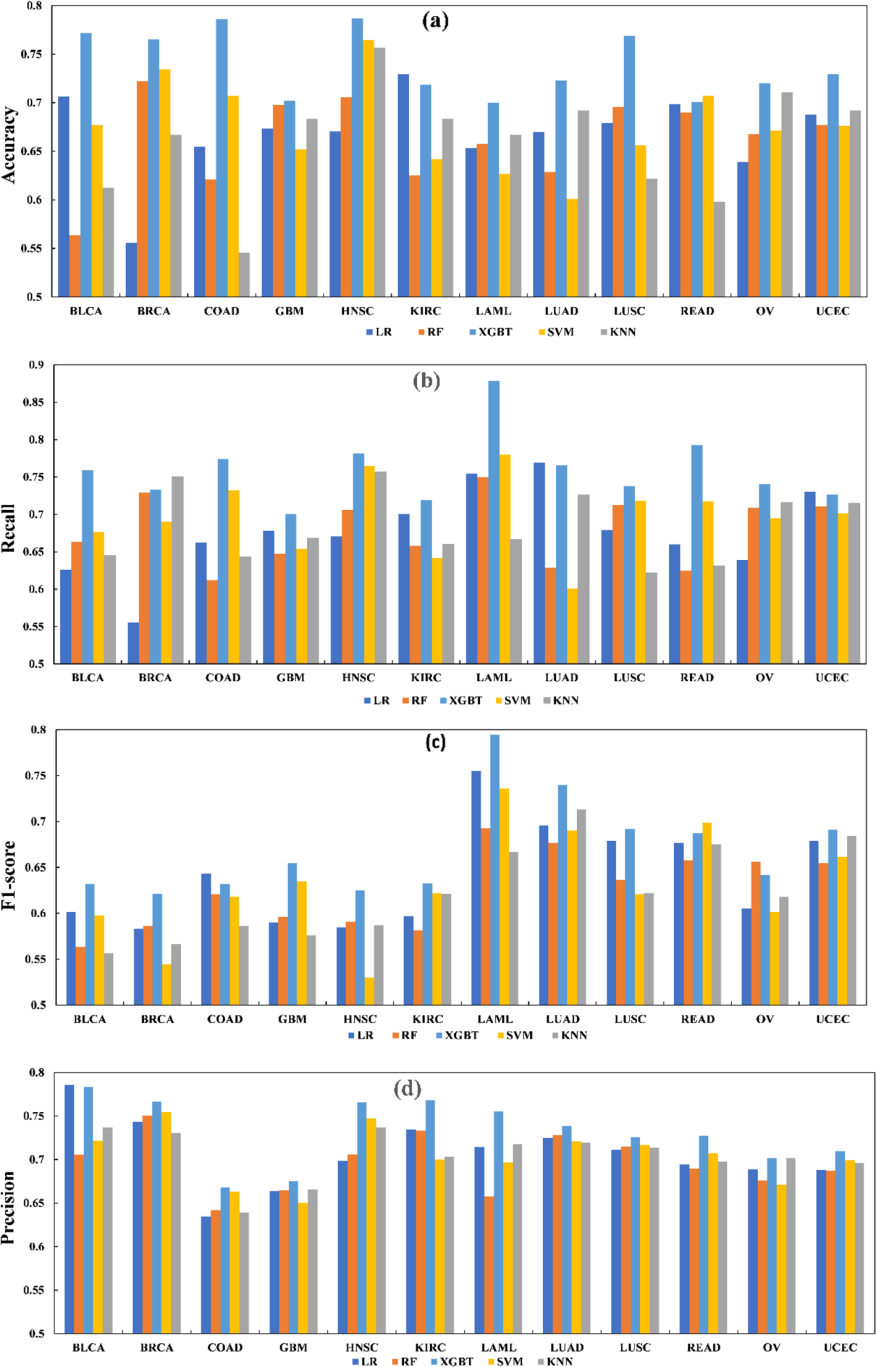
Comparison of four metrics of five machine learning algorithms in each cancer. The accuracy, Precision, Recall, and F1-score of the five algorithms were compared. In each figure, the X-axis represents each cancer type. Y-axis represents the value of Accuracy, Precision, Recall, and F1-score respectively

We also use five machine learning models to identify cancer driver genes among 12 cancers to discuss which model has good performance metrics under our framework. We use CGC as the gene standard set, combine the HINT + HI2012 network with mutation data, and integrate it into our framework to forecast possible cancer driver genes, similar to Pan-cancer. In 12 cancers, our framework analyzes four indicators of five models in each cancer. As shown in Fig. 3, it can be seen that the prediction model of XGBT is relatively good in each index. In the accuracy metric, XGBT outperforms almost every other algorithm, except in KIRC and COAD, where XGBT does not perform as significantly. In the recall metric, XGBT outperforms almost other algorithms, except in BRCA, UCEC, and LUAD, where XGBT does not perform as significantly. In the F1-score, it is obvious to see that XGBT is the most algorithm and superior to other algorithms, except that the effect of OV is not so obvious. In the precision metric, the XGBT method also outperforms other methods in most data sets.

### Impact of mutation data

**Fig. 4:**
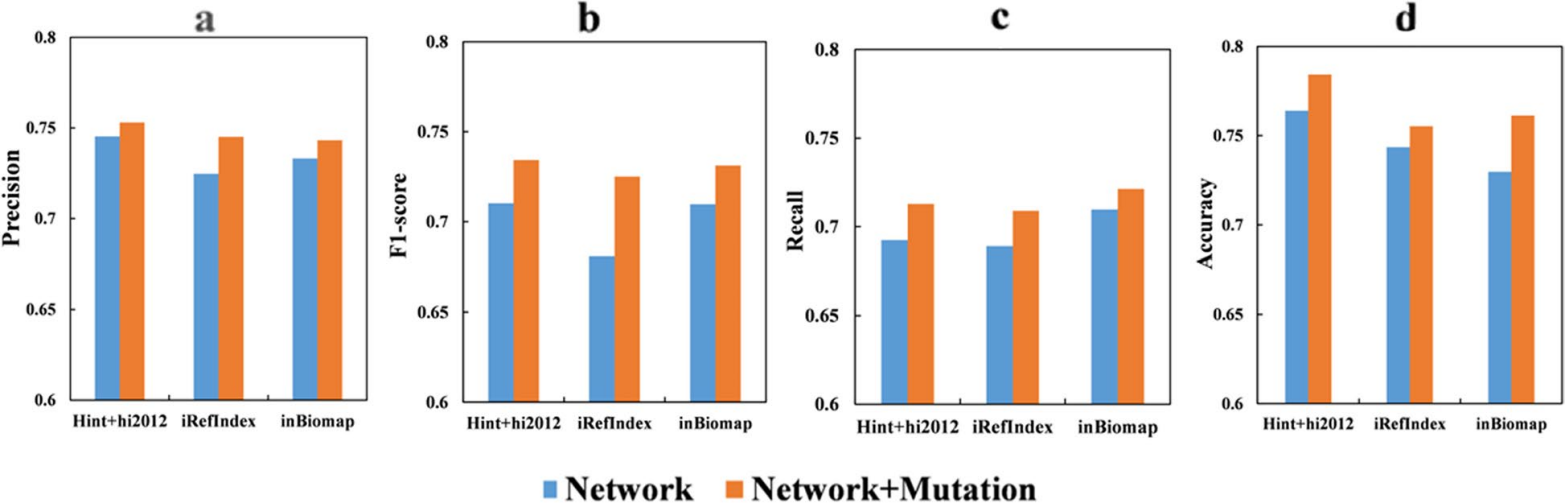
Under the optimal model XGBT model, the comparison of four indexes of data with and without mutation information

We detect driver genes from data with and without mutation information to discuss which is most effective. As important information to prioritize driver genes, we also study the impact of three individual networks on effectiveness. We take CGC as the optimal XGBT model under the gene standard set. As shown in Fig. 4, in our framework, the network with mutation features predicted four higher performance metrics than the network without mutation features. We find that mutation information is an important factor in promoting cancer development. By processing three networks, we find that the graph obtained by HINT + HI2012 contains fewer edges than the latter two networks, which makes our model more suitable for sparse networks. It can also be seen that the HINT + HI2012 network is also better than the other two networks under various indicators, although it is not very obvious. Note that the gene interaction network selected here is the same as the mutant genes in the experimental data set of Pan-cancer. Therefore, this does not mean that the original reference network is sparse. We find that driver genes are more likely to be detected in data with mutation information compared to data without mutation information.

### Comparison of driver gene detection methods

**Fig. 5:**
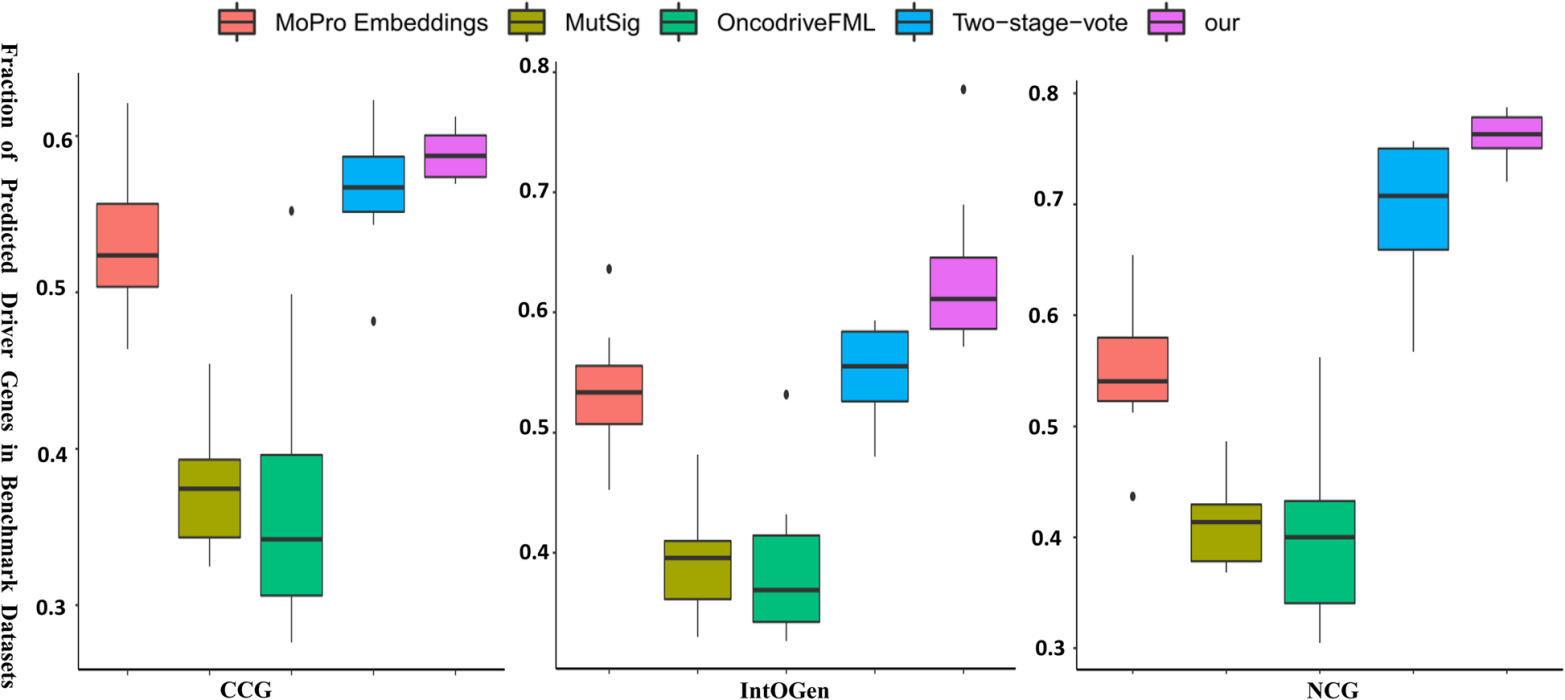
In three benchmark driver sets, we compared with four algorithms. Each group of panels corresponds to a particular benchmark driver set, and each box contains findings from each of the 12 different forms of cancer and represents one algorithm

In this paper, we compare our method with four excellent algorithms: MoProEmbeddings [17], MutSigCV [40], OncodriveFML [14], and Two-stage-vote [15]. Their prediction of driver genes came from DriverML. As shown in Fig. 5, it can be seen that the proportion of driver genes predicted in CGC, IntOGen, and NCG of 12 cancers in the TCGA database. Each panel represents a tool and is ranked according to the median score of the predicted driver genes in the above three gene standard sets. For a specific tool, the drivers of its prediction are different in different cancer types in three benchmark data sets. Our method ranks first. 59%, 61% and 78% of the predicted candidate driver gene belong to CGC, IntOGen, and NCG respectively. In CGC and NCG data sets, it can be seen that our method describes the discrete distribution of data in a relatively stable way. In the IntOGen dataset, there is an outlier in MoProEmbeddings, OncDriveFML, and our method. However, using MutSigCV and OncDriveFML, the predicted driver scores in the three databases are usually < 40%. In conclusion, our method successfully identifies a large number of cancer driver genes, and we believe it works well across a wide range of prevalent cancer types.

Our framework can find high-order neighbors in the network, and can also find gene pairs with long-distance but similar structures, which is more conducive to the identification of potential oncogenes. MutSigCV [40] method based on mutation frequency identification has been widely used in driver mutations and driving genes. OncodriveFML [14] uses the functional impact of gene mutations to reveal differences in coding and non-coding cancer drivers. The ensemble model is a Two-stage-vote [15] based on mutation information, gene networks, and a voting method that is created to find driver genes for 33 types of cancer. To enable supervised prediction, MoProEmbeddings [17] uses the knowledge of common cancer driver genes. Our framework outperforms other methods in some performance evaluations.

Our method is compared with four driver gene prediction algorithms using four metrics: Recall, AUPRC, F1-score, and Precision. As shown in Table 2, it can be seen that our method outperforms other methods in terms of recall metrics with the highest performance (0.746), followed by two-stage voting (0.739), MoProEmbeddings (0.436), and OncodriveFML (0.341). In the AUPRC metric, our method is the best performer, reaching (0.740), while the second-ranked Two-stage-vote method has a value of 0.658. In terms of index F1-score, our method is the best performer, reaching 0.679 higher. When comparing Precision, Two-stage-vote performs best, and the method ranks second in precision, the difference between the two is only 0.01.

**Table 2:** Comparison of five methods of performance evaluation

Method	Recall	F1-score	Precision	AUPRC	Algorithms
Our	**0.746**	**0.679**	0.781	**0.740**	XGBoost
MutSigCV	0.236	0.33	0.552	0.312	Logistic regressors
Two-stage-vote	0.739	0.635	**0.782**	0.658	Two-stage-vote
MoPro Embeddings	0.436	0.343	0.636	0.437	Gradient boosting
OncodriveFML	0.341	0.665	0.367	0.441	Functional impact

### Analysis of driver genes

**Fig. 6:**
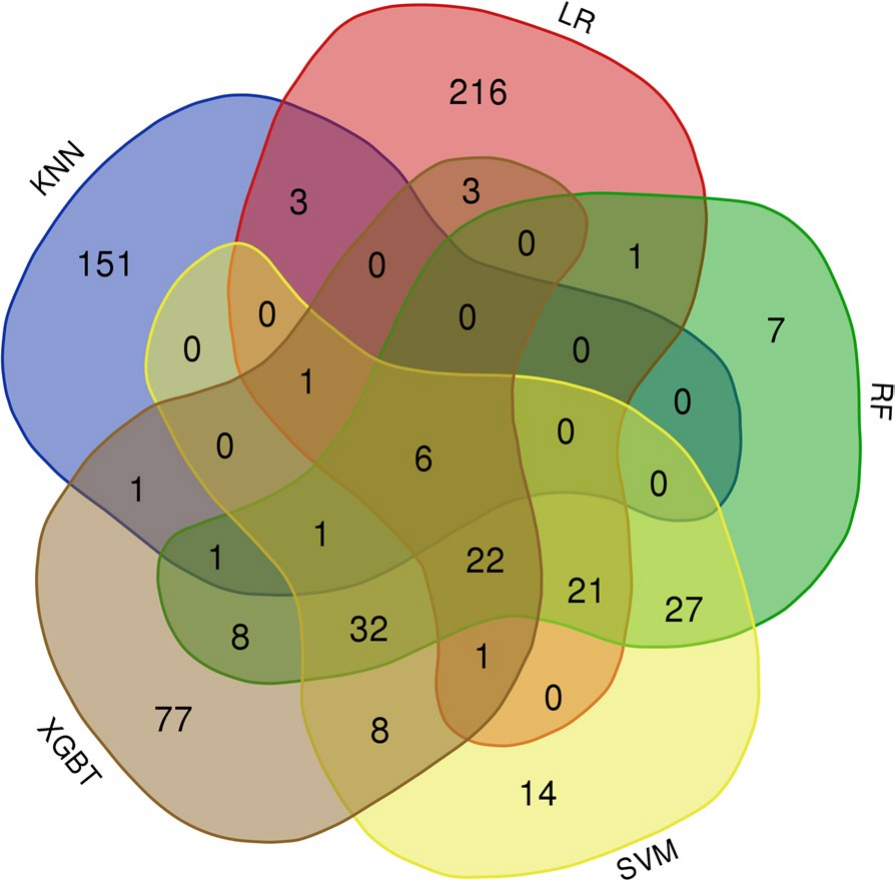
Venn diagram of known cancer genes predicted by different machine learning algorithms

It is important to identify potential cancer driver genes, which can also be predicted by several other methods. As the number of tools to identify these genes increases, the likelihood of predicting driver genes associated with cancer also increases. False positives in one tool may result in these genes being discarded by other identified tools. Five machine learning algorithms are used in this framework to detect known and unidentified cancer genes [41] (Table S2). We take the new genes predicted by these five machine learning methods and take the same ones and ranked them at the top. For these newly identified driver genes, using CarcerMine [42], a literature mining driver database, several significant genes are studied based on current literature reports. In general, each gene plays a different role, and even the dysregulation of certain essential genes can lead to cell death, so these genes play an even more important role in life activities. As shown in Fig. 6, it can be seen that the overlap of the five machine algorithms' predictions for cancer genes on the pan-cancer data.

The discovery of missense variants in PLEC that affect AF, combined with the recently identified variants of the muscle group genes MYH6 and MYL4, suggest that myocardial structure plays an important role in the pathogenesis of the disease [43]. ACVR1B expression levels are nominally significantly associated with emphysema distribution. It is associated with tumors through its interaction with activin A [44]. RASA1 expression is significantly reduced in KRAS wild-type colon cancer cells, indicating that miR-21 activates the RAS signaling pathway by downregulating RASA1 expression [45]. SMARCC2 is not among the CancerMine genes, but we find some research through a searchable comprehensive database GeneCards [46], which provides comprehensive information on all annotations and predictions of human genes. Frameshift alterations in colorectal and gastric cancers have been reported to cause the early arrest of amino acid synthesis of SMARCC2 protein, similar to the typical loss of function mutations. Surprisingly, the tumor-suppressive activity of SMARCB1 has been demonstrated, and this gene has been added to the CGC databases. To summarize, SMARCC2 needs more investigation as a potential cancer driver gene [47]. ZMYND8 is also involved in transcription regulation during normal cellular growth, which when disrupted increases cellular processes that lead to cancer start and development [48].

We also did positive control data from well-known driver genes found in different cancer types. We have selected three of these cancers for analysis. The well-known driver genes we identified from BRCA of breast cancer include AKT1, CDKN1B, ESR1, GATA3, MAP3K13, TP53, etc. Well-known driver genes identified from pancreatic cancer included AKT2, DAXX, FAT4, KRAS, etc. LUAD included ARAF, EED, GPC, TP53, etc. The rest of the cancers were also analyzed and can be seen in Table S3.

### Enrichment analysis

**Fig. 7:**
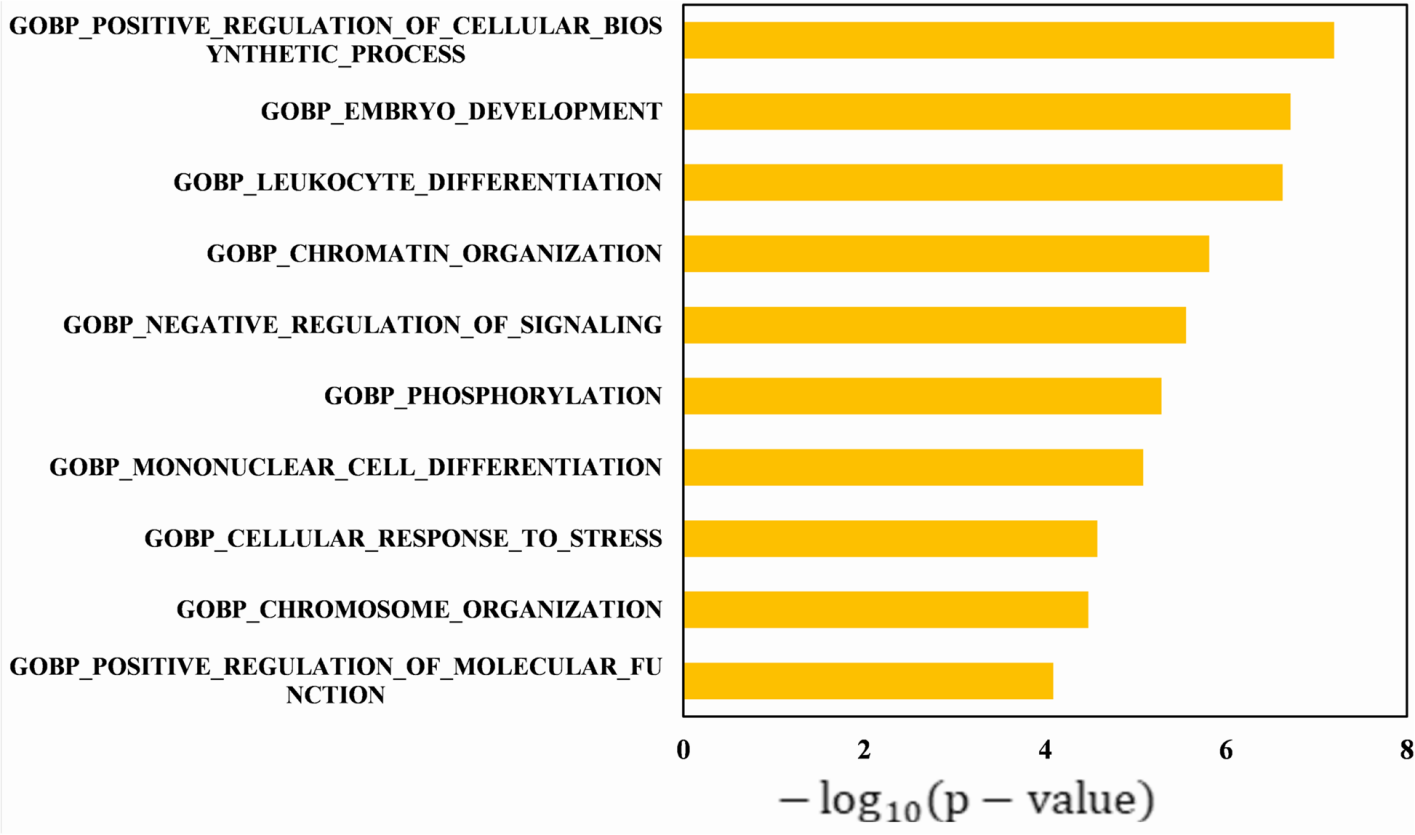
The 10 most significantly enriched pathways of GO pathways are ranked by *P*-value

**Fig. 8:**
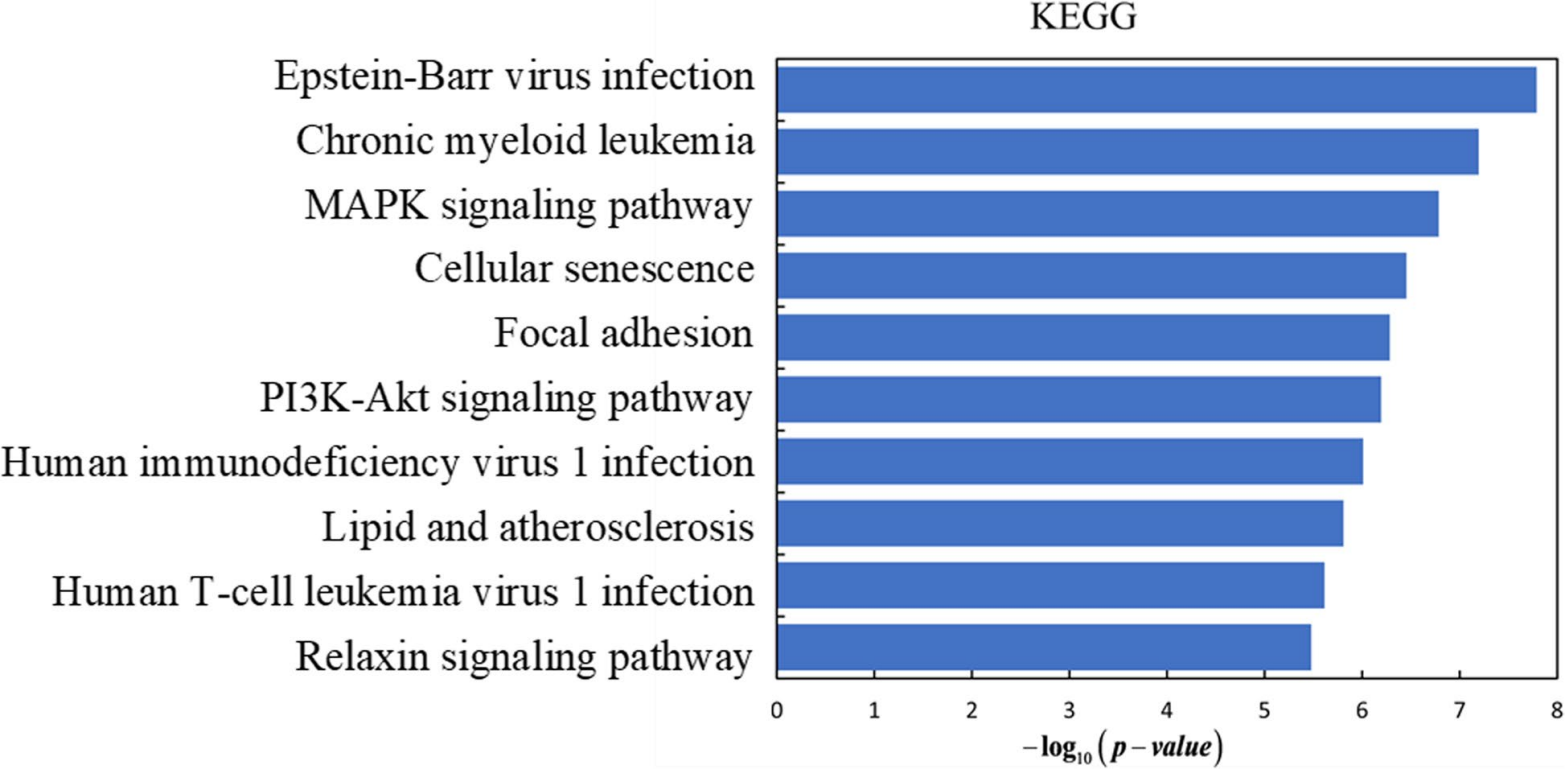
The 10 most significantly enriched pathways of KEGG pathways are ranked by *P*-value

In our framework, the top 100 genes in each method are mapped to GO terms such as molecular function (MF), cellular component (CC) and biological processes (BP), and pathways in KEGG, and statistically significantly enriched GO terms or pathways are detected and counted. The driver genes detected by five machine learning algorithms are analyzed by Gene Set Enrichment Analysis (GSEA) [49] (http://www.gsea-msigdb.org/gsea/msigdb/annotate.js) and Enrichr [50, 50] to investigate their statistical significance and biological relevance. We select the top 100 gene ontology terms with *P* value < 0.05 after each driver gene set enrichment analysis (Table S4 and Table S5). The driver gene sets predicted by five methods have 23 common GSEA gene ontology terms (Table S4), such as GOBP_PROGRAMMED_CELL_DEATH, GOCC_CHROMOSOME, GOBP_POSITIVE_REGULATION_OF_MOLECULAR_FUNCTION and so on. The driver gene sets predicted by five methods have 20 common Enrichr terms (Table S5), such as Epstein-Barr virus infection, Chronic myeloid leukemia, MAPK signaling pathway, and so on. We analyzed the predictive genes in the KEGG [51] data to understand the significantly altered metabolic pathways, which are particularly important in the mechanism study. As shown in Fig. 7 and Fig. 8, we rank the top 10 pathways in order of their P-values. And the p-value is transformed to be $- \log_{10} \left( {p - value} \right)$. Overall, the majority of prevalent gene ontology concepts are linked to cell death, cell differentiation, cellular proliferation, cell activation, the immune system, and other biological processes, all of which play essential roles in cancer formation.

## Conclusions

In order to detect potential cancer driver genes in cancer, we propose a network embedding framework that combines functional and structural information in this work. The mutation integration network is constructed by combining mutated genes with protein interaction networks using a network propagation algorithm. Using the struc2vec model to extract gene features from the mutation integration network, gene pairs with long distances but a similar structure can be found. Finally, machine learning algorithms are used to predict genes. Therefore, our framework takes into account more comprehensive information, including functional and structural information about genes, which is more conducive to identifying potential cancer driver genes. At the same time, we also compare and analyze three gene standard sets, three gene interaction networks, and various machine learning algorithms. In addition, our method outperforms other methods such as MoProEmbeddings, MutSigCV, and OncodriveFML. Our method can more accurately pinpoint potential cancer-causing genes.

However, our framework has some challenges. In future work, two aspects can be improved. On the one hand, our framework mainly uses the functional and structural features of genes, and can also take more features into account. On the other hand, in clinical practice, we can discuss the inclusion of more histological features to identify cancer driver genes in precision medicine and personalized medicine.

## Supplementary Information

**Additional file 1: Figure 1.** The parameter for network propagation genetic similarity of PPI network.. α=0.5 is selected as the best parameter for the network propagation algorithm, which has the highest precision. **Table 1.** Comparison of methods.

**Additional file 2.**

**Additional file 3.**

**Additional file 4.**

**Additional file 5.**

## Data Availability

There are no new data associated with this article. This data is from the TCGA website (https://portal.gdc.cancer.gov/) and the Catalogue of Somatic Mutations in Cancer (COSMIC) (https://portal.gdc.cancer.gov/) [23] and The pan-cancer is from reference [52]. In addition, the code of our are available at https://github.com/FengLi12/Our-code.

## Abbreviations

TCGA: The Cancer Genome Atlas
ICGC: International Cancer Genome Consortium
CGC: Cancer Gene Census
NCG: Network of Cancer Genes
IntOGen: Integrative Onco Genomics
BLCA: Bladder urothelial carcinoma
BRCA: Breast invasive carcinoma
COAD: Colon adenocarcinoma
GBM: Glioblastoma multiforme
HNSC: Head and neck squamous cell carcinoma
KIRC: Kidney renal clear cell carcinoma
LAML: Acute Myeloid Leukemia
LUAD: Lung adenocarcinoma
LUSC: Lung squamous cell carcinoma
READ: Rectum adenocarcinoma
OV: Ovarian serous cystadenocarcinoma
UCEC: Uterine corpus endometrial carcinoma
KNN: K-Nearest Neighbor
LR: Logistic Regression
XGBT: XGBoost
SVM: Support Vector Machine
RF: Random Forest
