# Supplementary material for: Network embedding framework for driver gene discovery by combining functional and structural information
Source: BMC Genomics. 2023 Jul 29;24:426. doi: 10.1186/s12864-023-09515-x (PMC10386255; doi:10.1186/s12864-023-09515-x)
Supplement: Supplementary file 1 — Additional file 1: Figure 1. The parameter for network propagation genetic similarity of PPI network.. α=0.5 is selected as the best parameter for the network propagation algorithm, which has the highest precision. Table 1. Comparison of methods. [file 12864_2023_9515_MOESM1_ESM.docx]

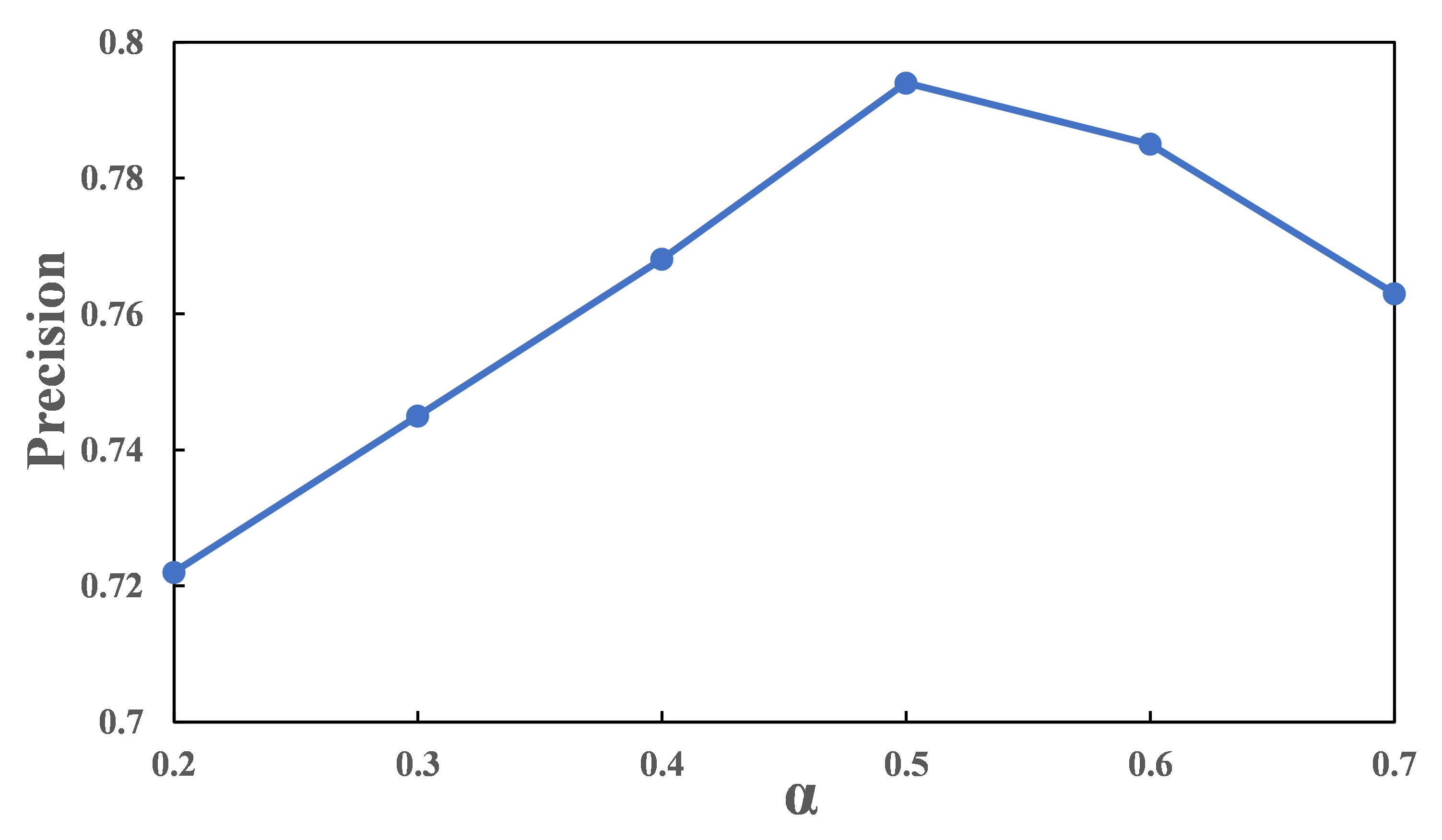


**Figure1**. The parameter for network propagation genetic similarity of PPI network.. α=0.5 is selected as the best parameter for the network propagation algorithm, which has the highest precision.

**Table1** Comparison of methods

| Method | F1-score | Recall | Precision | Accuracy | AUPRC |
| --- | --- | --- | --- | --- | --- |
| XGBT | 068 | **0.75** | **0.72** | **0.73** | **0.74** |
| Logistic regression | 0.66 | 0.59 | 0.73 | 0.64 | 0.70 |
| Random Forest | 0.70 | 0.71 | 0.37 | 0.68 | 0.64 |
| SVM | 0.31 | 0.60 | 0.62 | 0.63 | 0.69 |
| KNN | **0.75** | 0.59 | 0.77 | 0.66 | 0.70 |

In other metrics, XGBT is higher than the other methods, except in the F1-score, which is slightly lower than the other methods
